# Knowledge and Geo-Object Based Graph Convolutional Network for Remote Sensing Semantic Segmentation

**DOI:** 10.3390/s21113848

**Published:** 2021-06-02

**Authors:** Wei Cui, Meng Yao, Yuanjie Hao, Ziwei Wang, Xin He, Weijie Wu, Jie Li, Huilin Zhao, Cong Xia, Jin Wang

**Affiliations:** School of Resources and Environmental Engineering, Wuhan University of Technology, Wuhan 430070, China; yaomeng@whut.edu.cn (M.Y.); haoyuanjie@whut.edu.cn (Y.H.); zwei@whut.edu.cn (Z.W.); 2962575697@whut.edu.cn (X.H.); wwjie@whut.edu.cn (W.W.); Ljie@whut.edu.cn (J.L.); zhaohl2016@whut.edu.cn (H.Z.); 265107@whut.edu.cn (C.X.); 0121608900228@whut.edu.cn (J.W.)

**Keywords:** remote sensing images, semantic segmentation, geo-object prior knowledge, graph neural network

## Abstract

Pixel-based semantic segmentation models fail to effectively express geographic objects and their topological relationships. Therefore, in semantic segmentation of remote sensing images, these models fail to avoid salt-and-pepper effects and cannot achieve high accuracy either. To solve these problems, object-based models such as graph neural networks (GNNs) are considered. However, traditional GNNs directly use similarity or spatial correlations between nodes to aggregate nodes’ information, which rely too much on the contextual information of the sample. The contextual information of the sample is often distorted, which results in a reduction in the node classification accuracy. To solve this problem, a knowledge and geo-object-based graph convolutional network (KGGCN) is proposed. The KGGCN uses superpixel blocks as nodes of the graph network and combines prior knowledge with spatial correlations during information aggregation. By incorporating the prior knowledge obtained from all samples of the study area, the receptive field of the node is extended from its sample context to the study area. Thus, the distortion of the sample context is overcome effectively. Experiments demonstrate that our model is improved by 3.7% compared with the baseline model named Cluster GCN and 4.1% compared with U-Net.

## 1. Introduction

With the development of convolutional neural networks (CNNs), pixel-based semantic segmentation models have achieved impressive results in dealing with remote sensing images. These models execute convolution operations on pixels to aggregate information from areas covered by convolution kernels and attach semantic labels to each pixel [1,2,3,4]. However, these approaches cannot exploit high-level semantic information. Additionally, receptive fields in convolution are limited (generally 3 × 3) [5] and unevenly distributed [6], so it is hard to obtain effective contextual information.

To address the above problems, some approaches have been proposed. For example, the non-local method [7] calculates the feature similarity between each pixel and other pixels on the feature map. Therefore, the information of global pixels is integrated into the center pixel. Although the non-local method can more effectively use the context information of the sample, it is computationally expensive and lacks high-level semantics such as topological relationships of geo-objects.

Recently, with the development of graph neural networks, object-based semantic segmentation models that use GNNs have attracted increasing attention. Compared with pixel-based methods in remote sensing images, GNNs use geo-objects as nodes of the graph. Geo-objects are superpixels in which every object is a set of pixels with similar spectral and textural features. The transformation from remote sensing image into geo-objects can avoid salt-and-pepper effects and is beneficial to extracting features of spatial correlation effectively as well. However, the following problem urgently needs to be solved to apply GNNs in remote sensing recognition.

According to Tobler’s first law of geography, everything is related to everything else, but near things are more related to each other. However, there are some exceptions during feature aggregation in GNNs.

**Figure 1:**
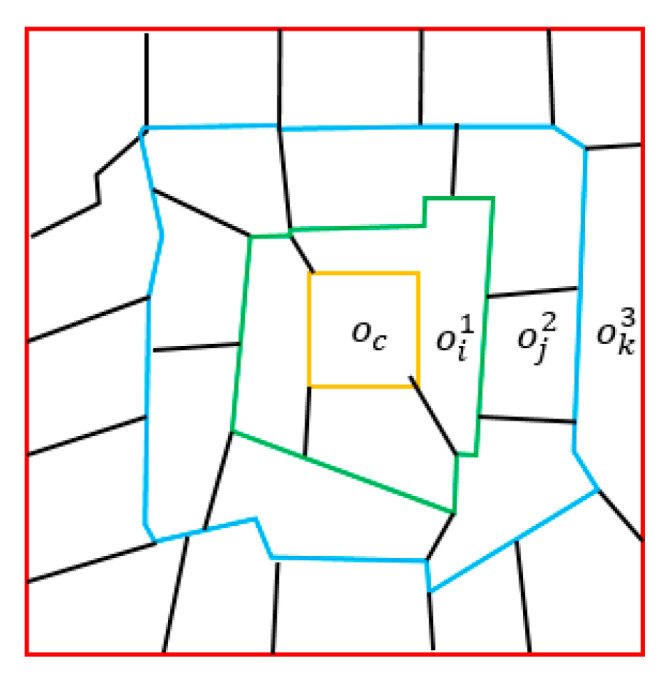
Demonstration of the center node and its neighbors with different spatial distances. The center node is $o_{c}$, $o_{i}^{1}$, $o_{j}^{2}$ and $o_{k}^{3}$ represent the neighboring nodes with spatial distances of 1, 2, and 3, respectively.

According to Figure 1, the aggregation of the center node can be expressed as Formula (1):

(1) $$o_{c}^{\prime }=\sum^{\;}w_{ci}^{1}*o_{i}^{1}+\sum^{\;}w_{cj}^{2}*o_{j}^{2}+\sum^{\;}w_{ck}^{3}*o_{k}^{3}$$

where w is the aggregation weight. As the perimeter of the outer polygon becomes larger, the number of adjacency objects becomes larger as well. If there is a large number of geographic objects with same category in the far distance, the aggregation of them may cause greater impact on the center node, even more than the nearby category. This may lead to the problem of “reversal of the first law of geography” and cause the misclassification of the center node. Samples facing this problem can be divided into two kinds:

(1) Samples with “different objects with the same spectrum”.

**Figure 2:**
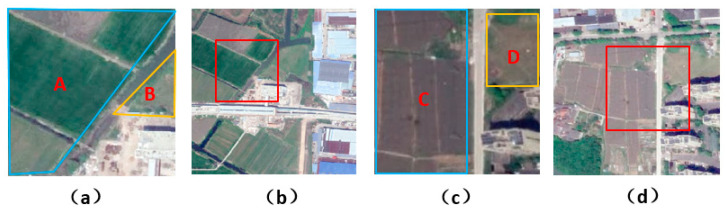
Examples of “different objects with the same spectrum”. (**a**,**c**) are two samples; (**b**,**d**) are corresponding surrounding environment of (**a**,**c**). In (**a**,**c**), the feature of city grass (in B) is similar to flat_field (in A). Meanwhile, in (**c**), the city_grass (in D) is similar to flat_field (in C).

“Different objects with the same spectrum” is a common phenomenon in remote sensing images, and nodes with similar features might be classified into different categories. During aggregation of center nodes, different nodes with the same spectrum are irrevelant neighbors. If there are a large number of these neighbors, these irrevelant neighbor nodes will have a greater impact on the center nodes, so the center node can be misclassified. Figure 2 demonstrates some of these samples.

As in Figure 2, flat_field and city_grass are “different objects with the same spectrum”, a large number of flat_fields will cause the misclassification of city_grass.

(2) Samples with “scene distortion”.

**Figure 3:**
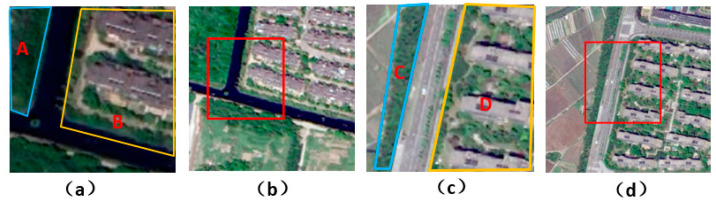
Demonstration of samples. (**a**,**c**) are samples with “scene distortion”. (**b**,**d**) show corresponding surrounding environment of (**a**,**c**). In (**a**), the forest object A is clipped improperly, and there is a large area of urban scene in B. Similarly, in (**c**), the urban scene D is near forest C.

During the process of making samples, improper sample clipping may result in incomplete neighbors of some objects in the sample; then, the surrounding environment of these objects in samples might be different from the actual situation. For example, forest and city_forest are all actually composed of trees, and the difference between them is that city_forest only exists in the urban scene. Due to improper clipping, there might be a large area of urban scene around the forest. As introduced before, objects in the urban scene will have a greater impact on the forest object. Then, this will lead to misclassification. Figure 3 demonstrates some of these samples.

Considering the above problems, the contextual information of one sample might be improper and limited; thus, the prior geographic knowledge of the entire study area is urgently needed. In this situation, the KGGCN model, which is an object-based remote sensing semantic segmentation model that integrates image features, spatial correlations, and prior knowledge with graph convolution, is proposed. This object-based method is free from the salt-and-pepper effect. Additionally, it can express high-level semantic information, for example, spatial correlations and prior geographic knowledge. Thereby, it can effectively recognize objects distributed by the above problem.

According to the above information, this paper has the following innovative aspects:

- This paper proposes a spatial correlation Recognition Module with mutual relation space to recognize spatial correlations between nodes and then automatically generate a spatial adjacency matrix.
- A mechanism of prior knowledge embedding that integrates the prior geographic knowledge of the study area by graph transformation is proposed.
- A semantic segmentation model of remote sensing images based on a graph neural network is designed and implemented. This model can organically integrate the spatial correlations of nodes with prior geographic knowledge so that the node’s receptive field is extended and the limitation of the sample context is broken through.

The next part of this paper is organized in the following sections: Section 2 discusses related work. Section 3 introduces the methods of our model. Section 4 is about the experiments. Section 5 analyses the model performances on specific samples. Section 6 analyses the performance of our model on the Hyperspectral Image Dataset. Finally, Section 7 expresses the conclusions.

## 2. Related Work

### 2.1. Geographic Object-Based Image Analysis (GEOBIA)

Different from the traditional pixel-based approach, GEOBIA is based on a geographic object and aims to divide remote sensing imagery into meaningful image-objects and then obtain information [8], as the object-based methods can provide richer semantic information and typological relations than pixel-based methods.

With the development of high-spatial resolution (H-res’) satellite sensors, it is more convenient to obtain high-resolution remote sensing images, which makes it urgent to propose effective methods of GEOBIA. Currently, the GEOBIA methods are widely applied in multi-scale studies [9,10,11], change analysis [12,13], and landslide detection [14]. Due to the nature of image analysis, GEOBIA benefits greatly from knowledge such as the work in [15], which surveys the kinds of urban problems from big data and discovers the knowledge of urban informatics. To facilitate effective knowledge exchange and management, the GEOBIA community has started to embrace ontologies and develop ontology-driven models [16] based on object-oriented remote sensing technology.

### 2.2. Graph Neural Network

CNN [17] has shown impressive ability to represent images and achieved great progress. However, these models fail to deal with non-Euclidean structure data such as social networks, chemical compounds, and knowledge graphs. Hence, GNN is proposed to conduct convolution on non-Euclidean data [18]. Among GNNs, the graph convolutional network (GCN) [19] plays an important role, which has been applied to many graph-based applications. However, it is still challenging in training a large-scale GCN model. Considering this problem, ref. [20] proposes a Cluster-GCN model, which samples the node blocks associated with the dense subgraph identified by the graph clustering algorithm, restricting the neighborhood search in the subgraph, and adds residual blocks in the graph convolution to achieve better performance in information aggregation. By reducing the amount of computation, this model is also suitable for a large graph process. The attention mechanism is significant in deep learning, and graph attention networks (GATs) [21] are proposed to focus on neighbor nodes’ features, which applies different weights to different nodes in a neighborhood and does not need costly matrix operation.

However, that the current GNN network directly stacks more layers can cause an over-smoothing problem, which can lead to the representations of GNN output nodes tending to be consistent. In this situation, the expressivity of the network is limited. To solve this problem, many researchers have conducted considerable explorations. Research such as [22] proposes a novel normalization layer that is based on a careful analysis of the graph convolution operator, which prevents all node embeddings from becoming too similar. Refs. [23,24] propose to use a jump connection and attention mechanism during graph convolution. Some other studies alleviate over-smoothing problems by deleting some edges in the graph; for example, ref. [25] proposes a DropEdge mechanism to randomly remove a certain number of edges from the input graph at each training epoch and reduces the convergence speed of over-smoothing or relieves the information loss caused by it.

Apart from these, combining prior knowledge with GNN models for vision tasks also attracts increasing attention. Ref. [26] constructs a graph by using co-occurrence of categories counting from the dataset to learn image features and explore their interactions via graph information propagation. Ref. [27] proposes a novel architecture called the Self-Constructing Graph (SCG), which can generate embeddings and construct the underlying graphs directly from the input features without relying on manually built prior knowledge graphs. Research such as [28] finds that the statistical correlations between object pairs and their relationships can improve performance in recognition and make prediction less ambiguous. To achieve this goal, it incorporates these statistical correlations into deep neural networks to facilitate scene graph generation by developing a knowledge-embedded routing network.

Recently, there have been relatively few studies on knowledge in the graph network. Additionally, the acquisition and utilization of knowledge in these methods are complex.

### 2.3. Remote Sensing with GNN

With the development of computer vision, remote sensing analyses are mostly based on deep learning algorithms. Traditional deep learning models for remote sensing analysis mostly use a full convolution structure [2,3,4]. However, these models are pixel-based so that it is difficult to extract high-level semantic information and typological relation of geo-objects. In this situation, the applications of GNNs in remote sensing have attracted increasing attention.

With the development of remote sensing technology, an increasing number of high-resolution remote sensing images have appeared. Some researchers have started to use GNNs to achieve remote sensing image analysis. Ref. [29] investigates the use of GCN in order to characterize spatial arrangement features for land use classification from high-resolution remote sensing images. The work [30] proposes a novel attention mechanism including horizontal and vertical directions and a graph convolution integration algorithm to achieve better performance on hyperspectral remote sensing image classification. Ref. [31] proposes a sampling technique, structure-aware sampling (SAS), which leverages the intra-class and global–geodesic distances between nodes and considers global information during message propagation. Recently, ref. [32] proposed a novel attention graph convolution network (AGCN) to perform superpixel-wise segmentation in big SAR imagery data. However, AGCN is prone to errors when segmenting some geo-objects with a small scale, such as rivers and roads.

In remote sensing images, the actual sizes of various geo-objects are quite different. Thus, understanding scale information [33] is essential for remote sensing image interpretation. Ref. [34] proposes a self-adaptive segmentation (SAS) method, which bridges the gap between the inherent scale and segmentation scale of each object. For better modeling of the multi-scale information of land-cover classes in remote sensing images, ref. [35] integrates high-dimensional multi-scale guided filter (MSGF) features with the superpixel-level guidance image. Ref. [36] applies thematic maps derived from image classification to improve multiscale segmentation and assist with scale selection. However, due to the rich semantics and complex topological relationship between geo-objects in remote sensing images, these methods cannot define the optimal scale in an unsupervised manner effectively.

Currently, some researchers start to integrate the geographic prior knowledge into remote sensing analysis. The work [37] proposes a simplified graph-based visual saliency model for airport detection in panchromatic remote sensing images, which introduces the concept of near parallelism for the first time and treats it as prior knowledge that can fully exploit the geometrical relationship of airport runways. The work [38] proposes to use prior knowledge provided by Volunteered Geographic Information (VGI) and extract the total extent of the roads using remote sensing images.

In summary, the combination of prior knowledge and geo-object-based graph convolutional network is a promising exploration in GEOBIA. On the one hand, the object-based method is able to alleviate the salt-and-pepper effects. On the other hand, the prior knowledge reflects the characteristic of the whole dataset; the integration of prior knowledge can expand the receptive field of the geo-object that needs to be analysed.

## 3. Methods

Unlike pixel-based image segmentation algorithms, we use an object-based graph convolutional algorithm. Image features are transformed into node features, and a spatial adjacency matrix is generated with correlation recognition. Then, the network will update the node features by integrating spatial adjacency matrix and prior knowledge. Finally, the updated node features are used for classification. The next sections will introduce the structure of the network and implementation details.

### 3.1. Network Structure

**Figure 4:**
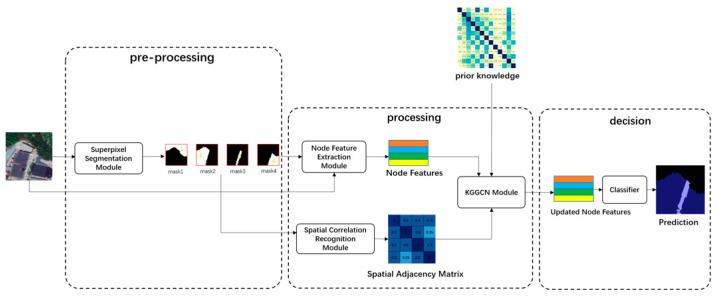
The structure of our approach. The input is the original image, and the output is the prediction.

Our network is composed of five modules, including the superpixel segmentation module, node feature extraction module, spatial correlation recognition module, KGGCN module, and classifier module, to carry out semantic segmentation on remote sensing images, as shown in Figure 4. The input is the original image, and the output is the prediction.

As shown in Figure 4, our approach is composed of five modules and can be divided into three steps: pre-processing, processing, and decision.

**Pre-processing**: It is important to explain that our model is not end-to-end, and the pre-processing step is before training the network. This step is composed of the superpixel segmentation module, the purpose of which is generating superpixel blocks and masks. First, the original image is divided into many superpixel blocks. Then, masks are generated according to the position of corresponding superpixel blocks in the image.

**Processing**: This step is composed of three modules: the node feature extraction module, spatial correlation recognition module and KGGCN module. The inputs of this step are the original image, masks, and prior knowledge. The outputs are updated node features, which incorporate geographic prior knowledge.

(1) The node feature extraction module extracts node features from the image. Inputs of this module are the original image and masks; outputs of this module are node features. First, the remote sensing image is extracted through a CNN to obtain the global feature. Then, masks are used to obtain the feature of each superpixel block. Finally, these features are passed through a global average pooling layer to obtain node features.
(2) The spatial correlation recognition module can recognize the spatial correlation of nodes and then generate the spatial adjacency matrix. Inputs of this module are masks, and the output of this module is a spatial adjacency matrix. First, features of two masks are extracted to obtain feature embedding of spatial correlation. Then, the embedded feature is decoded to obtain correlation of these masks. Finally, a spatial adjacency matrix is generated by traversing all pairs of masks and recognizing correlations.
(3) The KGGCN Module integrates graph convolution with prior knowledge to update node features. Inputs of this module are node features, a spatial adjacency matrix, and the prior knowledge. Outputs of this module are new node features. The KGGCN Module embeds prior knowledge into graphs for feature aggregation and then updates node features.

The details of these modules are introduced in Section 3.3, Section 3.4 and Section 3.5 in our paper, respectively.

**Decision**: This step is composed of a classifier. Inputs of this module are new node features from KGGCN Module. The output of this module is the classification result. The classifier is composed of a fully connected layer, which decodes node features to obtain predictions of nodes and the classification result.

### 3.2. Superpixel Segmentation Module

**Figure 5:**
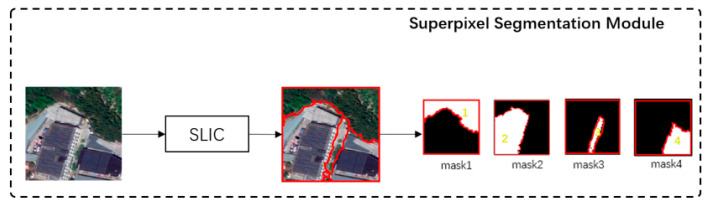
The structure of the superpixel segmentation module. The input is the image, and the outputs are masks.

The superpixel segmentation module aims to divide images into superpixel blocks and extract masks of them. The structure of this module is demonstrated in Figure 5.

In Figure 5, we use the simple linear iterative clustering (SLIC) [39] method to perform superpixel clustering on the remote sensing image and divide images into many superpixel blocks according to spectral and textural similarity. Then, the masks are established to reflect the position of the corresponding superpixel blocks in the image.

Details of this module are introduced next.

#### 3.2.1. Scale of Superpixel Segmentation

Understanding scale information [33] is crucial for remote sensing image interpretation. It is very important to determine the segmentation scale when using the SLIC algorithm to perform superpixel segmentation on the sample. In order to follow the principle that each superpixel block contains only a single category of pixels, ensuring the category label of superpixel block unique, we choose a small segmentation scale. Next, we will introduce the relative parameter settings of SLIC.

##### The Relative Parameter Settings of SLIC

The main parameters are k (number of clusters) and m (allows weighing the relative importance between colors). According to the following reasons, we finally set experience value (k = 15, m = 35) for small segmentation scale.

(1) The size of sample in our dataset is 224 × 224 with 0.59 m spatial resolution, with an actual distance about 134 metres. Therefore, the number of clusters does not need to be too large.
(2) The scale fits as closely as possible to the natural size of the small class of objects.
(3) Otherwise, the number of graph nodes in GCN should not be too large, avoiding very large computational consumption.

In order to better show the advantages of small-scale segmentation, we compared the segmentation between large scale and small scale on specific sample.

##### Comparison of the Segmentation between Large Scale and Small Scale

**Figure 6:**
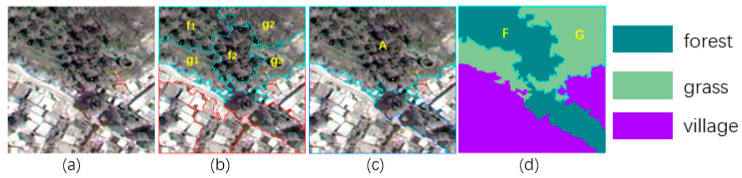
Comparison of the segmentation between two scales. (**a**) denotes original image; (**b**) denotes the result of small scale segmentation; (**c**) denotes the result of large scale segmentation; (**d**) denotes GT.

In order to illustrate the reason for choosing a small segmentation scale, we compared the small-scale segmentation (k = 15, m = 35) with a large-scale segmentation (k = 5, m = 20) on the same sample. The results are shown in Figure 6.

According to GT, the superpixel blocks f1 and f2 in (b) are forest, and superpixels g1, g2, and g3 in (b) are grass. Although large forest object F and large grass object G are divided into pieces, this still conforms to the principle that each superpixel block contains only a single category of pixels. However, the superpixel A in (c) contains two kinds of category pixels (grass and forest); the category label of A is not unique. Therefore, A is not suitable for effectively training the model.

#### 3.2.2. Establishment of Masks

**Figure 7:**
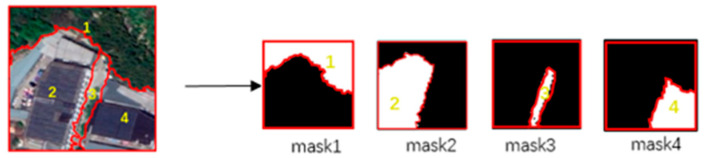
Demonstration of masks. Mask 1–4 reflects the position of superpixel block 1–4 in image respectively.

After small-scale superpixel segmentation, the image is divided into superpixel blocks. The size of the masks of the superpixel block is 224×224, which is the same as image. In each mask, pixels within the range of the superpixel block are set as 1 and all other pixels are set as 0. Figure 7 demonstrates the establishment of masks.

### 3.3. Node Feature Extraction Module

**Figure 8:**
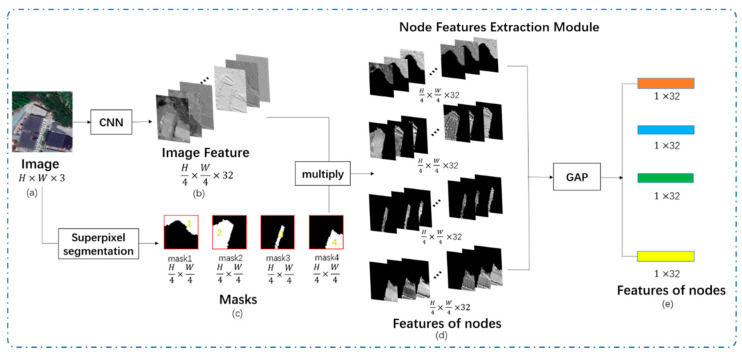
The structure of the node feature extraction module. (**a**) is the original image; (**b**) is the image feature; (**c**) are resized masks; (**d**) are two-dimensional features of nodes; (**e**) are one-dimensional features.

The structure of the node feature extraction module is demonstrated in Figure 8.

As shown in Figure 8, CNN is used to extract the image feature (b). The size of (b) is $\frac{H}{4}\times \frac{W}{4}\times 32$, where 32 is the feature dimension.

Masks are obtained from the superpixel segmentation module. To reflect positions of superpixel blocks in the image feature, masks are resized to $\frac{H}{4}\times \frac{W}{4}$.

The features extracted by masks are used as the features of nodes in the graph network. Masks are multiplied by the image feature to obtain features of nodes.

Features of nodes are passed through a global average pooling (GAP) layer and changed from two-dimensional features (d) to one-dimensional feature vectors (e).

### 3.4. Spatial Correlation Recognition Module

Inspired by the mutual relation space in [40], we design a spatial correlation recognition module. The spatial correlation recognition module aims to recognize the spatial correlations of nodes to construct the spatial adjacency matrix. The details of this module will be introduced next.

#### 3.4.1. Mutual Relation Space

In traditional relation space, $\left(o_{1},\;p_{1},\;o_{2}\right)$ is object triples, where $o_{1}$ and $o_{2}$ are objects and $p_{1}$ is the relation between them. The feature of object triples is used for recognizing relation.

$p_{1}$ is the relation from $o_{1}$ to $o_{2}$, and $p_{2}$ is the relation from $o_{2}$ to $o_{1}$. The relation of $p_{1}$ and $p_{2}$ is denoted as mutual interaction.

Visual relationships have great potential to be learned better with the knowledge from the mutual interactions between paired objects. However, traditional relation space fails to exploit the mutual interaction between objects. Therefore, we construct a mutual relation space, and the mutual interaction of relations is considered during feature extraction.

#### 3.4.2. Spatial Adjacency Matrix

Before introducing the spatial adjacency matrix, we firstly introduce the means of node feature aggregation in GCN [19], as shown in Formula (2).

(2) $$f_{i}^{\prime }=\overset{N}{\underset{j=1}{\sum }}a_{ij}f_{j}$$

$f_{i}^{\prime }$ is the feature of node *i* after the update, and $f_{j}$ is the feature of neighbor node *j*. $a_{ij}$∈$A^{0}$, $A^{0}$ is the spatial adjacency matrix, as shown in Formula 3.

(3) $$A^{0}=\begin{array}{ccc} a_{11} & \cdots & a_{1N}\\ \vdots & \ddots & \vdots \\ a_{N1} & \cdots & a_{NN} \end{array},\;A^{0}\in R^{N\times N}$$

In Formula (3), $A^{0}$ is the spatial adjacency matrix and the value in the adjacency matrix represents the spatial correlation between two nodes. For example, $a_{ij}$ represents the spatial correlation between node *i* and node *j*.

- If mask *i* and mask *j* are directly adjacent, the spatial correlation of node *i* and node *j* is ‘closely next to’, corresponding to the value $a_{ij}=0.5$ in the adjacency matrix;
- If *i* and *j* are separated by one node, the spatial correlation of *i* and *j* is ‘next to’, $a_{ij}=0.25$;
- If *i* and *j* are separated by two nodes, the spatial correlation of *i* and *j* is ‘near’, $a_{ij}=0.125$;
- If there are more than two nodes separating *i* from *j*, the spatial correlation of *i* and *j* is ‘far from’, $a_{ij}=0$.

According to the definition of the adjacency matrix, the correlation of nodes is the relationship between one node and another node. In order to generate the spatial adjacency matrix, the spatial correlation recognition module needs to recognize the spatial correlation between two nodes.

#### 3.4.3. Structure of the Spatial Correlation Recognition Module

**Figure 9:**
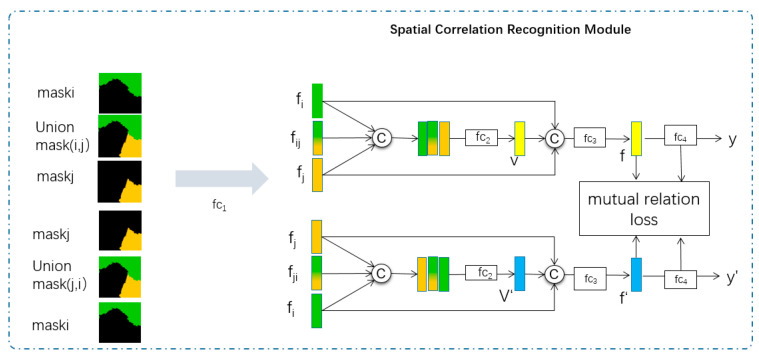
The framework of spatial correlation recognition module. The input of this module is pairs of masks, and the output is the recognized spatial correlation between masks. The $fc_{k}$ (k = 1, 2, 3, 4) represent different fully connected layers. *C* represents the concatenation operator to concatenate tensors in a specific axis.

The spatial correlation recognition module aims to effectively recognize spatial correlations in mutual relation space, as shown in Figure 9.

This module extracts features of masks and union masks. Then, these features are used to obtain the feature embedding of spatial correlation. Finally, the embedded features are used for correlation recognition.

The processes of this module are denoted as Algorithm 1, as follows
**Algorithm 1. For recognizing the spatial correlation.****Input:** $mask_{i}$ of superpixel block *i*, $mask_{j}$ of superpixel block *j*.**Output:** spatial correlation between $mask_{i}$ and $mask_{j}$ Begin // use mask to extract feature of $mask_{i}$ $f_{i}\leftarrow fc_{1}\left(mask_{i}\right);$ // $fc_{1}$ is a fully connected layer1 // use mask to extract feature of $mask_{j}$ $f_{j}\leftarrow fc_{1}\left(mask_{j}\right);$ // $fc_{1}$ is a fully connected layer1 // obtain union mask of $mask_{i}$ and $mask_{j}$ $mask_{ij}\leftarrow union\left(mask_{i},mask_{j}\right);$ // union is the operation of directly add by pixels // use union mask to extract feature of mask pair $f_{ij}\leftarrow fc_{1}\left(mask_{ij}\right);$ // $fc_{1}$ is a fully connected layer1 // obtain feature embedding of correlation $\left(i,\;p_{1},\;j\right)$ $V\leftarrow fc_{2}\left(concat\left(f_{i},\;\;f_{ij},\;\;f_{j}\right)\right);\;$//$fc_{2}$ is a fully connected layer2 $f\leftarrow fc_{3}\left(concat\left(f_{i},\;\;V,\;\;f_{j}\right)\right);//\;fc_{3}$ is a fully connected layer3 // obtain feature embedding of correlation $\left(j,\;p_{2},\;i\right)$ $V^{\prime }\leftarrow fc_{2}\left(concat\left(f_{j},\;\;f_{ij},\;\;f_{i}\right)\right);//\;fc_{2}$ is a fully connected layer2 $f^{\prime }\leftarrow fc_{3}\left(concat\left(f_{j},\;\;V^{\prime },\;\;f_{i}\right)\right);//\;fc_{3}$ is a fully connected layer3 // recognize spatial correlation between *i* and *j* $y\leftarrow argmax(fc_{4}\left(f\right));$ $\;//\;fc_{4}$ is a fully connected layer4 // recognize spatial correlation between *j* and *i* $y^{\prime }\leftarrow argmax(fc_{4}\left(f^{\prime }\right));$ $//\;fc_{4}$ is a fully connected layer4 Return y, $y^{\prime }$; End

This algorithm is the feature extraction process used for correlation recognition, and feature extraction is optimized by using mutual relation loss.

#### 3.4.4. Mutual Relation Loss

As shown in Formula (6), the mutual relation loss function is composed of loss1 (cross-entropy loss) and loss2 (margin loss calculates the similarity of f and $f^{\prime }$).

(4) $$\mathrm{loss}1=-\frac{1}{N\left(N-1\right)}\overset{N\left(N-1\right)}{\underset{i}{\sum }}y_{i}*\log y_{i}$$

(5) $$\mathrm{loss}2=\max\left(0,\left(0.5-l2(f\right)-l2\left(f^{\prime }\right)\right)$$

(6) loss=loss1+loss2

In above formulas, f and $f^{\prime }$ are feature embeddings of spatial correlation, as described in Algorithm 1. Loss1 is used for the training module to predict accurate spatial correlations. Loss2 is used to optimize the process of feature extraction.

### 3.5. KGGCN Module

As discussed in Section 1, directly using spatial correlations during feature aggregation fails to effectively solve problems of “the reversal of the first law of geography”. Prior knowledge that expresses characteristics from all samples in the study area is urgently needed. Therefore, the KGGCN Module uses the co-occurrence probability of various categories as prior knowledge, embeds prior knowledge into graphs for feature aggregation, and then extends the receptive field from a single sample to all samples in the study area. This section introduces the co-occurrence matrix and structure of the KGGCN Module.

#### 3.5.1. The Co-Occurrence Matrix

The co-occurrence matrix represents the probability of pairs of categories occurring in the same sample and describes the statistical characteristics from all samples in the study areas by calculating the frequency of two categories occurring in the same sample.

In our dataset, categories include flat_field, landslide, grass, waterbody, village, road, highway, city, terraces, strip_field, city_grass, forest, and city_forest. $M\in R^{C\times C}$ represents the co-occurrence matrix used to describe co-occurrence between categories, and *C* is the number of classes. The following formulas explain the calculation of co-occurrence probability. Class *α* and *β* influence each other; thus, the co-occurrence probability of *α* and *β* is bidirectional. $m_{\alpha \beta }$ represents the probability that class *β* appears near class *α*, and $m_{\beta \alpha }$ represents the probability that class *α* appears near class *β*. If $m_{\alpha \beta }$ is the direct co-occurrence probability, then $m_{\beta \alpha }$ is the corresponding reverse co-occurrence probability.

(7) $$m_{\alpha \beta }=\frac{n_{\alpha \beta }}{n_{\alpha }}$$

(8) $$m_{\beta \alpha }=\frac{n_{\beta \alpha }}{n_{\beta }}$$

**Figure 10:**
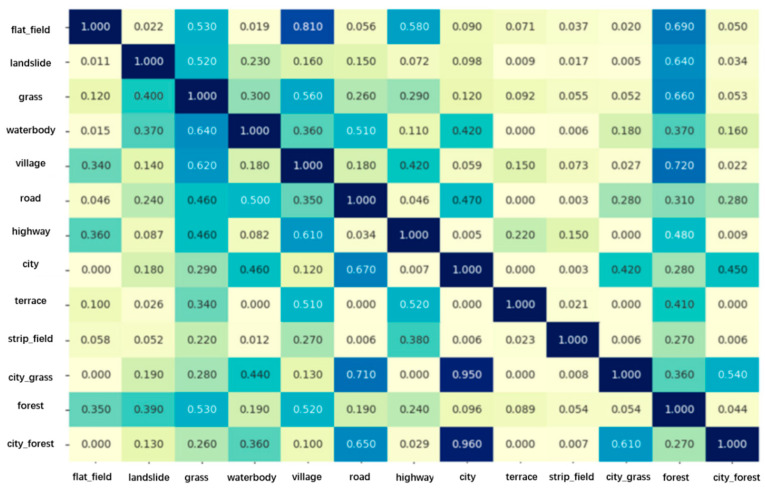
The co-occurrence matrix. Values in this matrix indicate the co-occurrence probabilities of various categories.

In Formula (7), $n_{\alpha \beta }$ represents the number of samples that simultaneously include class *α* and class *β*, $n_{\alpha }$ represents the number of samples that include class *α*, and $m_{\alpha \beta }$ equals to $n_{\alpha \beta }$ divided by $n_{\alpha }$. $m_{\beta \alpha }$ is calculated similarly in Formula (8). After calculating the co-occurrence probability between all classes, the co-occurrence matrix is used as prior knowledge, as shown in Figure 10.

In Figure 10, $m_{city,city\_grass}$ = 0.42 and $m_{city_{grass},\;city}$ = 0.95. It is easy to notice that city is most likely to occur near city_grass, because we denote grass as city_grass only if there is a city nearby. However, there may be no city_grass near city. Therefore, $m_{\alpha \beta }\neq m_{\beta \alpha }$.

#### 3.5.2. Structure of KGGCN Module

**Figure 11:**
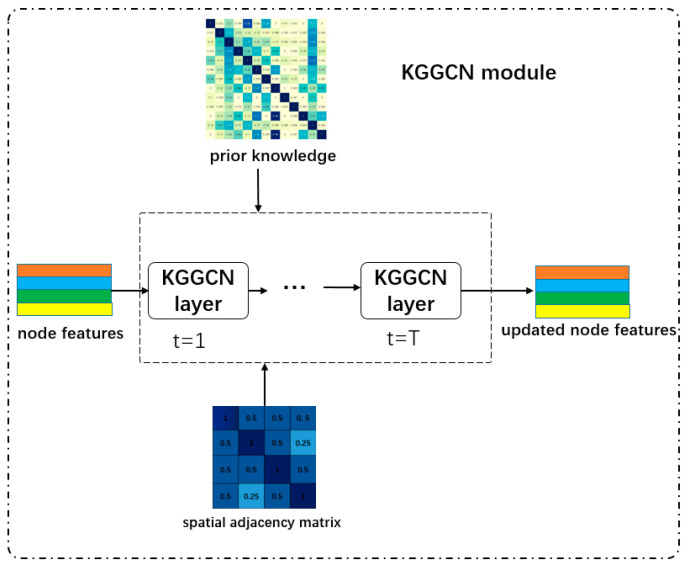
The structure of KGGCN module. T is the number of KGGCN layers.

The KGGCN Module belongs to the processing step of our model. The inputs of KGGCN Module include prior knowledge, node features, and the spatial adjacency matrix. Prior knowledge is the co-occurrence matrix. Node features are the outputs of the node feature extraction module. The spatial adjacency matrix is the output of the spatial correlation recognition module. Outputs of the KGGCN module are the updated node features. The structure of KGGCN module is shown in Figure 11.

In Figure 11, the KGGCN module is composed of two KGGCN layers (T = 2) with the same structure:

(1) The first KGGCN layer. Inputs of this layer are node features (obtained from the node feature extraction module), the spatial adjacency matrix (obtained from the spatial correlation recognition module), and prior knowledge (the co-occurrence matrix). The output is updated node features of the first layer.
(2) The second KGGCN layer. Inputs of this layer are updated node features of the first layer, the spatial adjacency matrix (obtained from the spatial correlation recognition module), and prior knowledge (the co-occurrence matrix). The output is updated node features of the second layer.

##### The Structure of the KGGCN Layer

**Figure 12:**
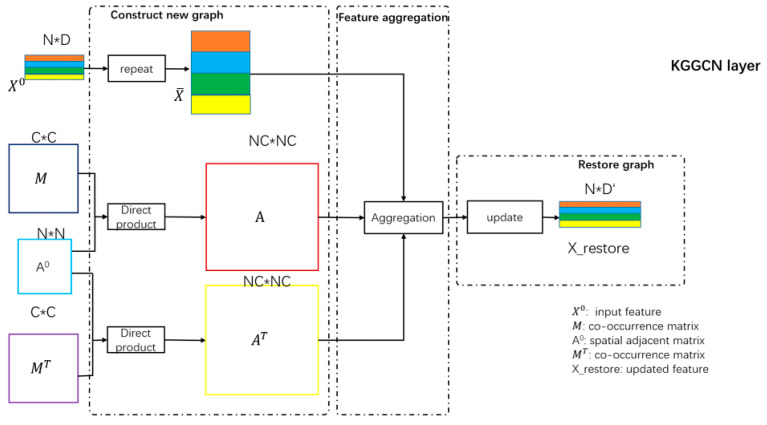
Structure of KGGCN layer. The input of the KGGCN layer includes four parts: $X^{0}$ denotes the node features in the original graph, $X^{0}\in R^{N\times D}$, where *N* is the number of nodes, and *D* is the dimension of the feature. M denotes the matrix of the co-occurrence probability, and $M\in R^{C\times C}$, where *C* is the number of categories. $M^{T}$ denotes the transpose of M. $A^{0}$ denotes spatial adjacency matrix, and $A^{0}\in R^{N\times N}$.

The purpose of the KGGCN layer is to integrate prior knowledge into graph convolution. The structure of the KGGCN layer is shown in Figure 12.

As demonstrated in Figure 12, the structure includes three parts: constructing a new graph, feature aggregation, and restoring the new graph. The purpose and implementation of these parts will be introduced next.

##### Mechanism of Prior Knowledge Embedding Based on Graph Transformation

Before the introduction of the KGGCN layer, the idea of prior knowledge embedding is explained first. In our model, the co-occurrence probability of various categories is prior knowledge. M is the matrix of co-occurrence probability, as introduced in Section 3.5.1, and $M\in R^{C\times C}$, where *C* is the number of categories. One specific sample is expressed as original graph $G_{ori}$ in Formula (9).

(9) $$G_{ori}=\left(V_{ori},E_{ori}\right)$$

$V_{ori}$ is the set of nodes that is superpixel blocks obtained from one sample; $E_{ori}$ is the set of edges that represent spatial correlations of nodes obtained from the spatial correlation recognition module, as in Formulas (10) and (11).

(10) $$V_{ori}=\left\{v_{i},i\in \left\{1,\cdots ,\;N\right\}\right\}$$

(11) $$E_{ori}=\left\{e_{ij},i,j\in \left\{1,\cdots ,\;N\right\}\right\}$$

In Formula (11), $e_{ij}=a_{ij}$, where $a_{ij}$ represents the spatial correlations of nodes. It is important to notice that the prior knowledge is a general knowledge.

While training the graph convolution network with $G_{ori}$, the category of nodes is unknown, and the matrix of co-occurrences expresses the relation of categories, so it is unable to directly embed prior knowledge into graph convolution. To address this problem, we propose a mechanism of prior knowledge embedding. Therefore, to realize the embedding of knowledge in the training process, we traverse all nodes in the original graph and consider the case in which a node is any class in *C* categories. Thus, $V_{ori}$ is duplicated based on the number of categories.

(12) $$G_{new}=\left(V_{new},E_{new}\right)$$

**Figure 13:**
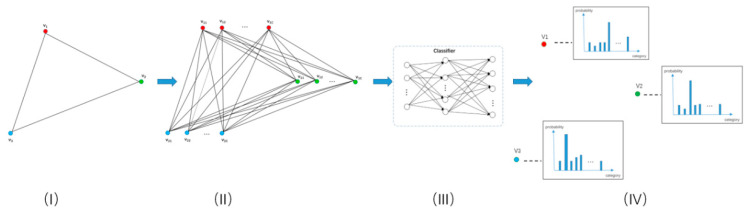
Process of prior knowledge embedding. (**I**) is the structure of the original graph $G_{ori}$; (**II**) is the structure of the new graph $G_{new}$ with prior knowledge embedding, as expressed in Formula (12); (**III**) is a classifier for classifying nodes, which is a simple multi-layer perceptron (MLP). (**IV**) is the restoration of $G_{new}$ after classifying nodes.

In Figure 13, from (I) to (II), $G_{new}$ is constructed by duplicating the nodes of $G_{ori}$ based on the number of categories, as shown in Formula (13) and (14), where N is the number of nodes in $G_{ori}$ and C is the number of categories.

(13) $$V_{new}=\left\{v_{i\alpha },i\in \left\{1,\;\ldots ,\;N\right\},\;\alpha \in \left\{1,\;\ldots ,C\right\}\right\}$$

(14) $$E_{new}=\left\{e_{\left(i\alpha ,j\beta \right)},i,j\in \left\{1,\;\ldots ,\;N\right\},\;\alpha ,\beta \in \left\{1,\;\ldots ,\;C\right\}\right\}$$

In Formulas (13) and (14), $v_{i\alpha }$ denotes node $v_{i}$ with category α, $v_{j\beta }$ denotes node $v_{j}$ with category β, and $e_{\left(i\alpha ,j\beta \right)}$ is the edge of $\;v_{i\alpha }$ and $v_{j\beta }$.

As explained in Section 3.5.1, the co-occurrence relation between categories is bidirectional, so edges in new graph are bidirectional as well. Direct edges represent the effect of neighboring nodes on the center node, and reverse edges represent the effect of center nodes on neighboring nodes.

Thus, $e_{\left(i\alpha ,j\beta \right)}=a_{\left(i\alpha ,j\beta \right)}\;or\;{a^{\prime }}_{\left(i\alpha ,j\beta \right)}$, as expressed in Formula (15) and (16).

(15) $$a_{\left(i\alpha ,j\beta \right)}=a_{ij}\times m_{\alpha \beta },\;m_{\alpha \beta }\in M$$

(16) $${a^{\prime }}_{\left(i\alpha ,j\beta \right)}=a_{ij}\times m_{\beta \alpha },\;m_{\beta \alpha }\in M$$

In Formula (15) and (16), $a_{\left(i\alpha ,j\beta \right)}$ denotes a direct edge that multiplies the spatial correlation by the direct co-occurrence probability, and ${a^{\prime }}_{\left(i\alpha ,j\beta \right)}$ denotes a reverse edge that multiplies the spatial correlation by the reverse co-occurrence probability.

Thus, co-occurrence probability as prior knowledge is embedded into the graph network by combining with spatial correlations. Then, node features will aggregate in $G_{new}$ to consider spatial correlations along with the co-occurrence probability.

From (II) to (IV), $G_{new}$ is restored to $G_{ori}$ with updated node features. In this process, nodes in $G_{new}$, each of which is generated by duplicating the same node in $G_{ori}$ for C times, are concatenated. Therefore, the updated node features will contain the information of all categories. Then, these node features are projected into a hidden dimension. After classification, the information of irrelevant categories in node features will be removed, and the restoration from $G_{new}$ to $G_{ori}$ is accomplished.

The implementation of prior knowledge embedding is introduced in the next section.

##### Processes of Prior Knowledge Embedding

**Figure 14:**
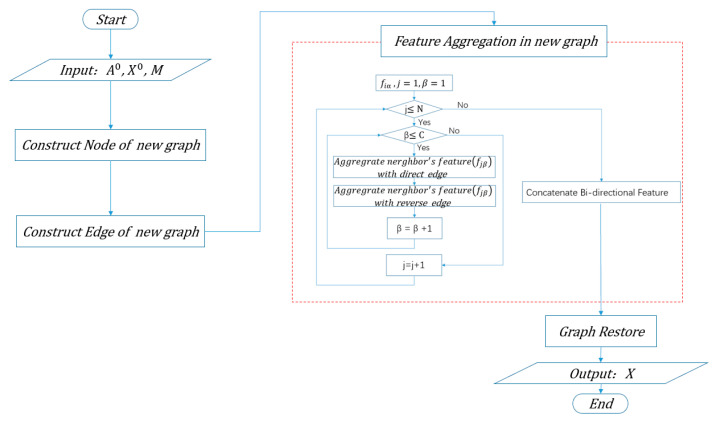
Flow chart of knowledge embedding. The inputs include a spatial adjacency matrix $A^{0}$, node features $X^{0},$ and co-occurrence matrix M. The output is updated node features X after knowledge embedding.

To better explain the details of the process, we use a flowchart in Figure 14 to express our idea.

According to the process in Figure 14, the implementation of the above processes includes four main steps: constructing the nodes of the new graph, constructing the edges of the new graph, feature aggregation in the new graph, and graph restoration.

**Step 1: Construct nodes of the new graph**

$X^{0}=\left\{f_{i},i\in \left\{1,\ldots ,N\right\}\right\}$ is a set of all node features in the original graph, where $f_{i}$ is the feature of node $v_{i}$. As mentioned before, we duplicate the feature of $v_{i}$ C times to obtain $\left\{f_{i1},f_{i2},\ldots ,f_{ic}\right\}$ corresponding to new nodes $\left\{v_{i1},v_{i2},\cdots ,v_{ic}\right\}$. This step is shown in Formula (17).

(17) $$X^{0}={\left[\begin{array}{c} \begin{array}{c} f_{1}\\ f_{2} \end{array}\\ \begin{array}{c} \vdots \\ f_{N} \end{array} \end{array}\right]}^{N\times D},\;\;\bar{X}=repeat\left(X^{0},C\right)={\left[\begin{array}{c} \begin{array}{c} \begin{array}{c} \begin{array}{c} f_{11}\\ f_{12} \end{array}\\ \vdots \\ f_{1C} \end{array}\\ f_{21} \end{array}\\ \vdots \\ f_{NC} \end{array}\right]}^{NC\times D}$$

In Formula (17), $f_{N}$ is the feature of the *N*-th superpixel blocks with dimensions of *D*, and $X^{0}\in R^{N\times D}$ are the features of nodes in the original graph. $\bar{X}\in R^{NC\times D}$ are features of all nodes in the new graph obtained by repeating the process C times from the original graph, so that the number of nodes in the new graph is N×C.

**Step 2: Construct edges of the new graph**

New edges are obtained by the direct product operation between spatial correlations of nodes and the co-occurrence probability of categories. As mentioned in the ’mechanism of prior knowledge embedding’ section, the constructed edges of the new graph include bidirectional edges:

(a) Construct direct edges of the new graph

As shown in Formula (18), A represents all direct edges and is obtained by the direct product of $A^{0}$ and M.

(18) A=A0⊗M=a11⋯a1N⋮⋱⋮aN1⋯aNNN×N⊗m11⋯m1c⋮⋱⋮mCC⋯mCCC×C=a11M⋯a1NM⋮⋱⋮aN1M⋯aNNMN×N=a(11,11)⋯a(11,NC)⋮⋱⋮a(N1,11)⋯a(NC,NC)NC×NC

In Formula (18), $A=\left\{a_{\left(i\alpha ,j\beta \right)}\right\}$, where i,j = 1, 2, …, N, α,β = 1, 2, …, *C*. After the direct product is constructed, the direct edges of all nodes in the new graph are generated.

(b) Construct reverse edges of the new graph

Similar to direct edges, the construction of reverse edges is a direct product of $A^{0}$ and $M^{T}$. $M^{T}\in R^{C\times C}$ is the transpose of M, and $\;A^{T}=A^{0}⊗M^{T}$ is executed to represent all reverse edges.$\;A^{T}=\left\{{a^{\prime }}_{\left(i\alpha ,j\beta \right)}\right\}$.

After generating new nodes and new edges, the new graph is constructed. Then, prior knowledge is integrated with the spatial adjacency matrix for aggregation.

**Step 3: Feature aggregation in the new graph**

As shown in Figure 14, $f_{i\alpha }$ denotes the feature of the center node $v_{i\alpha }$, and $v_{i\alpha }\in V_{new}$. It is important to note that feature aggregation includes two processes. The first process is feature aggregation with direct edges, and the second process is feature aggregation with reverse edges. After the loop operation, aggregated features with direct edges and aggregated features with reverse edges are concatenated to obtain the center nodes’ integrated features:

(a) Aggregate neighbors’ feature $\left(f_{j\beta }\right)$ with direct edges.

Feature aggregation with direct edges represents the effect of neighbor nodes to center node, as shown in Formula 19.

(19) $$f_{i\alpha }^{direct}=\sum_{j=1}^{N}\sum_{\beta =1}^{C}a_{\left(i\alpha ,j\beta \right)}\cdot f_{j\beta }\;$$

In Formula (18), $f_{i\alpha }^{direct}$ is the aggregated feature of node $v_{ic}$ with direct edges, and the aggregation includes twice traverse. The first is single node with all categories, β = 1, …, *C*. The second traverse is in all nodes, *j* = 1, …, *N*. Then obtain $f_{i\alpha }^{direct}\in R^{1\times D}$, *i* in 1, …, *N*.

(b) Aggregate neighbors’ feature $\left(f_{j\beta }\right)$ with reverse edges.

Feature aggregation with direct edges represents the effect of the center node on neighboring nodes. This operation is expressed in Formula 20, similar to Formula (19).

(20) $$f_{i\alpha }^{reverse}=\overset{N}{\underset{j=1}{\sum }}\sum_{\beta =1}^{C}{a^{\prime }}_{\left(i\alpha ,j\beta \right)}\cdot f_{j\beta }$$

In Formula (20), $f_{i\alpha }^{reverse}$ is the aggregated feature of node $v_{i\alpha }$ with reverse edges. Then, $f_{i\alpha }^{reverse}\in R^{1\times D}$ is obtained, *i* in 1, …, *N*.

(c) Concat Bi-directional Feature

After feature aggregation of the bi-edges, the aggregated features are concatenated in Formula (21). $f_{i\alpha }$ is the aggregated feature of node $v_{i\alpha }$.

(21) $$f_{i\alpha }=concat\left(f_{i\alpha }^{direct},f_{i\alpha }^{reverse}\right)$$

After concatenation, aggregated feature $f_{i\alpha }$ is obtained, where $f_{i\alpha }\in R^{1\times 2D}$, with *i* in 1, …, *N*. The aggregated feature contains the effect of center nodes on neighboring nodes and the effect of neighboring nodes on center nodes.

**Step 4: Graph Restoration**

As introduced before, $G_{new}$ needs to be restored to $G_{ori}$. Firstly, new graph nodes that are generated by duplicating from nodes in the original graph for C times are concatenated, as shown in Formula (22).

(22) $$X_{\_restore}=\left\{f_{i}\right\}=\left\{concat\left(f_{i1},f_{i2},\cdots ,f_{ic}\right)\right\},i=1,\;2,\;\cdots ,N$$

In Formula (22), $f_{i}$ is the feature of node $v_{i}$, $\left(f_{i1},f_{i2},\cdots ,f_{ic}\right)$ is a set of feature components of $f_{i}$, and $X_{\_restore}\in R^{N\times 2CD}$ represents node features after concatenation.

Then, $X_{\_restore}$ is projected to the output dimension with trainable parameters as shown in Formula (23).

(23) $$X_{\_new}=X_{\_restore}*W_{GCN\;}$$

In Formula (23), $W_{GCN}$ $\in R^{2CD\times D^{\prime }}$ represents trainable parameters used to project features into the hidden dimension, and $X_{\_new}\in R^{N\times D^{\prime }}$ represents node features where $D^{\prime }$ is the output dimension of the node features.

Finally, all nodes are classified, and the redundant information of irrelevant categories is removed through the classifier shown in Formula (24).

(24) $$\mathrm{Output}=\varphi \left(FC\left(f_{1},f_{2},\cdots ,f_{N}\right)\right)$$

In Formula (24), $\left(f_{1},f_{2},\cdots ,f_{N}\right)$ represents the updated node features, FC represents a fully connected layer with SoftMax that obtains probability vector for prediction, and φ represents the argmax operation that selects the category with the highest probability as the predicted category.

### 3.6. Discussion

Unlike the traditional aggregation mechanism, our KGGCN model proposes a mechanism of prior knowledge embedding before graph convolution. Then, after feature projection, node features integrate spatial correlations and prior geographic knowledge.

### 3.7. Depth of Network and Loss Function

#### 3.7.1. Depth of Graph Neural Network

The Cluster-GCN model has three graph convolution layers and is our baseline model. The first two layers of graph convolution aim to update nodes’ feature. Every node in the graph is traversed as a center node, neighboring node features are aggregated with the center node, and then graph convolution is performed to update the center node feature. The third graph convolution layer is used for classification, and the output of this layer is the classification probability matrix of nodes.

The KGGCN Module in our model is composed of two KGGCN layers and one fully connected layer (fc layer). The two graph convolution layers are used to embed prior knowledge into the graph to extract node features with geographic prior knowledge, and the fc layer aims to achieve node feature classification.

#### 3.7.2. Loss Function

Loss functions in our model can mainly be divided into two different parts.

As introduced in Section 3.4, one part of loss is used for spatial correlation learning in the spatial correlation recognition module. This part is composed of cross-entropy loss and margin loss (Formulas (4)–(6)). Cross entropy loss is used to classify spatial correlations, and margin loss aims to improve the performance of module feature extraction with positive and inverse relationships in the relative position. This part of the loss is used for pretraining so that the parameters of spatial correlation recognition will not be optimized in the next modules.

Another part of loss is used for the KGGCN Module. This part of the loss is cross-entropy loss, which aims to calculate the error between the node prediction probability vector and the node label one-hot vector, as shown in Formula (25).

(25) $$\mathrm{loss}=-\frac{1}{N}\overset{N}{\underset{i}{\sum }}y_{i}*\log y_{i}$$

## 4. Experiments

### 4.1. Study Area and Introduction of Samples

This study involves Wenchuan County, Sichuan Province, and the surrounding study area, with dimensions ranging from North 30°28′41″ to North 30°32′29″ and East 114°22′42″ to East 114°28′11″. We select a total of 1680 patches from the research area to obtain enough samples to train and validate the network. We randomly divided all samples into a training set with 1280 samples and a validation set with 400 samples. Each sample is composed of a remote sensing image with a size of 224 × 224, a manually classified ground truth image with the same size of 224 × 224, and an object segmentation image from a remote sensing image processed by an open-source superpixel algorithm.

### 4.2. Experimental Environment and Hyper-Parameters

We conduct experiments with the hardware environment of RTX 3080 GPU and 64G RAM. Meanwhile, the software environment includes ubuntu16, cuda10.1, and pytorch 1.6.0.

Experiments involve a traditional semantic segmentation model named U-Net, the Cluster-GCN model, used as the baseline of graph convolution, and our model (KGGCN). According to previous experiments, the feature dimension of the last layer in U-Net is set as 512, batch size is 32, the learning rate is 3e-4, and the total training number of epochs is 300; the baseline model Cluster-GCN needs graph convolution to be performed three times, the output dimension of the two first graph convolution layers is 64, and the output dimension of the last graph convolution layer is 13 (number of classes). Our KGGCN model requires two graph convolution layers and one fully connected layer. The output dimensions of the graph convolution layers are all 64, and the dimension of the fully connected layer is 13 (number of classes). For Cluster-GCN and KGGCN, batch sizes are all 1, learning rates are 1 × 10^−3^, the numbers of training epochs are 500, and dropout rates are 0.2.

### 4.3. Loss Curves

**Figure 15:**
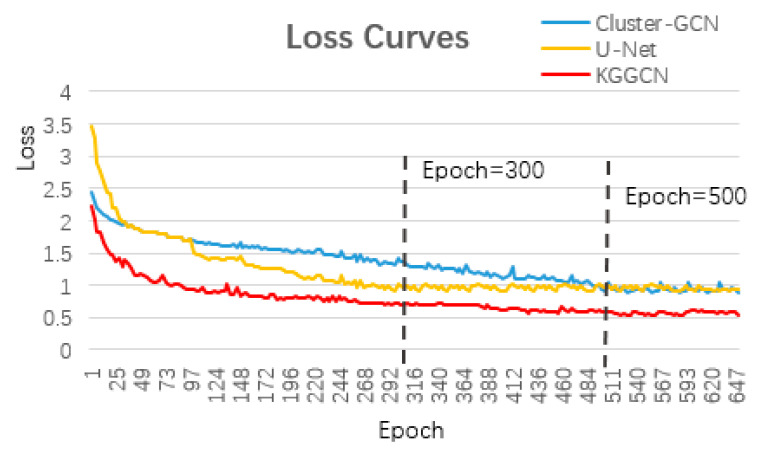
Loss curves of the three models: Cluster-GCN, U-Net, and KGGCN. The x-axis is the training epoch and the y-axis is loss.

We use the Adam optimizer in the three models and the loss curves are demonstrated in Figure 15.

The loss curve expresses the convergence tendency of the model. As the number of iterations increases, the loss decreases and finally tends to be stable. U-Net basically converges after training for up to 300 epochs. Cluster-GCN and our KGGCN both converge when they reach 500 training rounds. Therefore, fewer training rounds are needed for U-Net to converge than for Cluster-GCN and KGGCN to converge. For U-Net, the number of the training epoch is 300. For Cluster-GCN and KGGCN, the numbers of training epochs are 500. When all models converge, the losses of U-Net and Cluster-GCN are nearly 0.95, and the loss of KGGCN is approximately 0.5. Therefore, the KGGCN model is more effective than the U-Net and Cluster-GCN models in the semantic segmentation of remote sensing images.

### 4.4. Analysis of Total Accuracy

**Figure 16:**
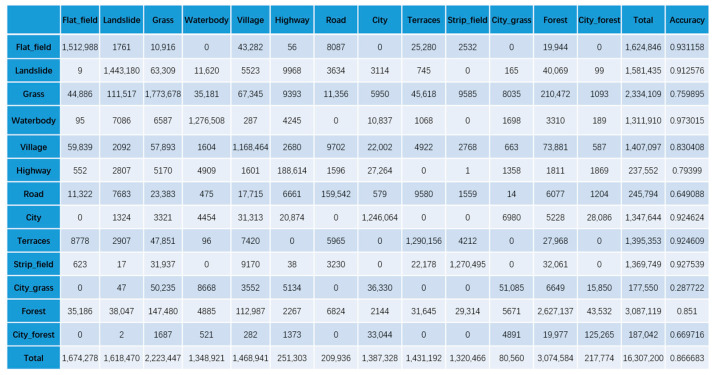
The pixel-based confusion matrix of the U-Net model.

**Figure 17:**
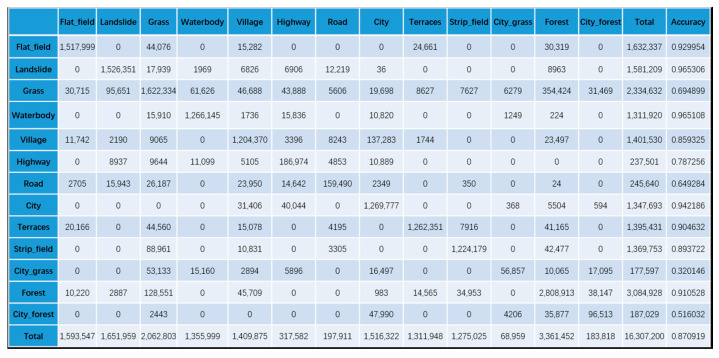
The pixel-based confusion matrix of the Cluster-GCN model.

**Figure 18:**
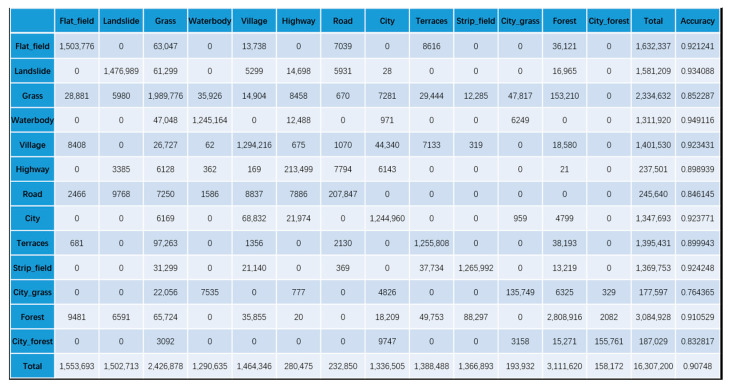
The pixel-based confusion matrix of the KGGCN model.

**Figure 19:**
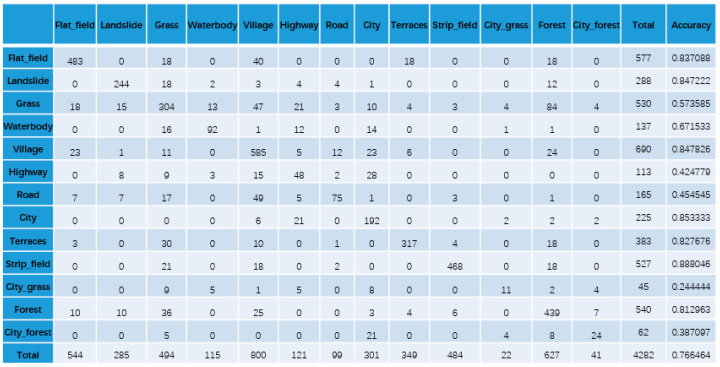
The object-based confusion matrix of the Cluster-GCN model.

**Figure 20:**
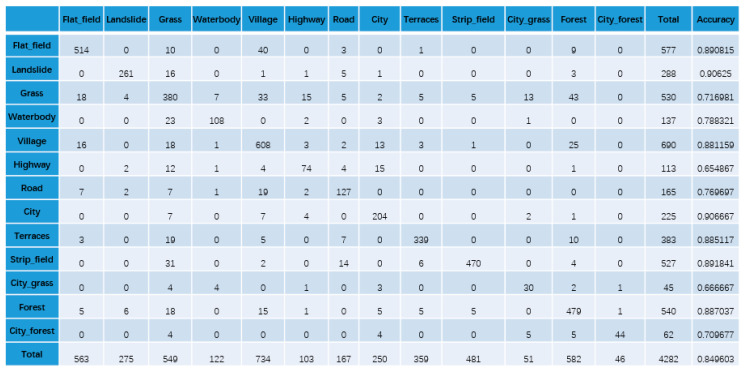
The object-based confusion matrix of the KGGCN model.

To confirm the effectiveness of our KGGCN model, we compare the classification confusion matrices of U-Net, Cluster-GCN, and our KGGCN model using our dataset. Figure 16, Figure 17, Figure 18, Figure 19 and Figure 20 are confusion matrices. To compare with U-Net, the object-based confusion matrix is converted to a pixel-based confusion matrix.

According to the confusion matrices in Figure 16, Figure 17, Figure 18, Figure 19 and Figure 20, the pixel segmentation accuracies of U-Net, Cluster-GCN, and the KGGCN (our model) are 0.8667, 0.8709, and 0.9074, respectively. The object classification accuracy of Cluster-GCN is 0.7665, while that of the KGGCN (our model) is 0.8496. U-Net is a pixel-based model, so it does not have object classification accuracy. According to the pixel segmentation performance, our model’s accuracy is 4.1% higher than that of U-Net and 3.7% higher than that of Cluster-GCN. According to the object classification performance, our model’s accuracy is 8.3% higher than that of Cluster-GCN. Thus, our model achieves better performance in the semantic segmentation of remote sensing images.

Additionally, we further compare the three networks in other classification metrics, and the results are shown in Table 1.

As shown in Table 1, the KGGCN model also has a significantly better accuracy, mIOU, Kappa, and F1-score than the other models. The bold indicates the result of our model.

Table 2 shows the pixel-based accuracies of categories in three models.

According to Table 2, we can find that:

- U-Net and Cluster-GCN obtain bad results when classifying city_grass; their accuracies are 0.288 and 0.320, respectively. According to the confusion matrices in Figure 16 and Figure 17, in all samples where city_grass was classified incorrectly, more than 50% of the city_grass samples were classified as grass. However, the KGGCN accuracy for city_grass classification is 0.764, and only a few city_grass samples are misclassified as grass.
- U-Net and Cluster-GCN also obtain bad results when classifying city_forests; their accuracies are 0.670 and 0.516, respectively. According to the confusion matrices in Figure 16 and Figure 17, among all samples that were classified incorrectly, most city_forest samples were classified as forest. In Figure 18, KGGCN’s accuracy for city_forest classification is 0.833, and only few city_forest samples are misclassified as forest.

Additionally, we add the performance and system resource requirements of the three networks, as shown in Table 3. Params represents the size of the model. Mem represents the training GPU memory consumption. FLOPs represents the calculation amount. Inf time represents the inference speed of model, which can refer to the execution times of models.

As shown in Table 3, the KGGCN model has obvious advantages in model size, resource occupation, calculation amount, and inference speed.

In a word, compared with U-Net and Cluster-GCN, KGGCN achieves better performance in semantic segmentation in remote sensing images.

## 5. Analysis of Typical Samples

**Figure 21:**
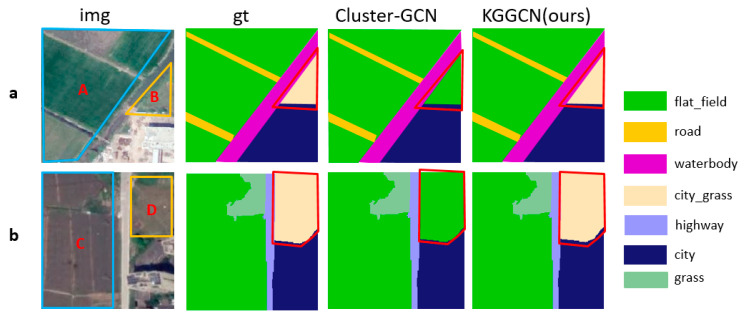
The result of two models in samples with “different objects with the same spectrum”. In (**a**), city_grass (B) is misclassified as flat_field in Cluster-GCN and classified correctly in KGGCN. In (**b**), city_grass (D) is misclassified as flat_field in Cluster-GCN and classified correctly in KGGCN.

As expressed in Section 1, the problem of “the reversal of the first law of geography” cannot be solved by directly using spatial correlation as edges. By integrating prior knowledge with spatial correlations, these problems can be effectively solved. To further analyze the advantage of the KGGCN model, we compare the result of Cluster-GCN (baseline) and KGGCN (ours) in atypical samples. According to Section 1, samples facing with “the reversal of the first law of geography” can be divided into two kinds:

One is samples with “different objects with the same spectrum”; superpixel blocks with similar textures and spectra might be different classes. Samples are demonstrated in Figure 21.

**Figure 22:**
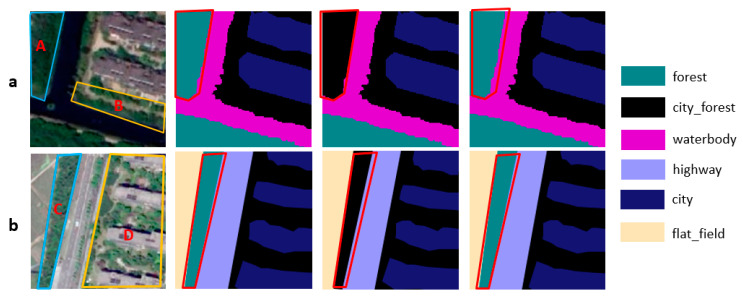
The result of two models in samples with “scene distortion”. In (**a**), forest (A) is misclassified as city_forest in Cluster-GCN and classified correctly in KGGCN. In (**b**), forest (C) is misclassified as city_forest in Cluster-GCN, classified correctly in KGGCN.

The other is samples with “scene distortion”. Some geo-objects in these samples are clipped improperly. Samples are demonstrated in Figure 22.

Then, we compare the performance of KGGCN model with Cluster-GCN by analyzing specific sample and discuss the advantage of embedding prior knowledge.

(1) Analysis of a sample with “different body with the same spectrum object”

**Figure 23:**
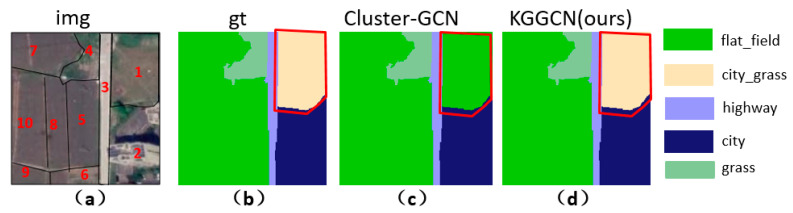
Demonstration of one sample. (**a**) is raw remote sensing image, and superpixel blocks are marked in the image; (**b**) is the ground truth of this image; (**c**) is the prediction of Cluster-GCN; (**d**) is the prediction of KGGCN.

As shown in Figure 23, node 1 is city_grass, misclassified as flat_field in Cluster-GCN and classified as city_grass correctly in KGGCN. The two models both obtain correct classifications for other nodes.

The next analysis comes from two parts: nodes’ feature and the effectiveness of prior knowledge embedding.

(a) Nodes’ feature—foreign body with the same spectrum.

**Figure 24:**
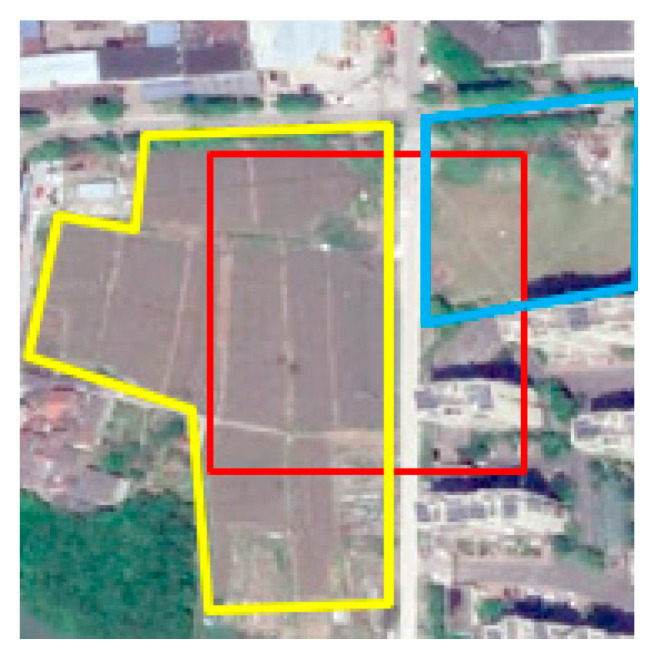
Large-scale image of the sample. The red box represents the sample in Figure 23, city_grass (in blue box) is misclassified as flat_field (in yellow box) by Cluster-GCN, and City_grass and flat_field are ‘different objects with similar spectrum’.

As shown in Figure 24, flat_field is similar to city_grass in spectral feature. During the feature update of city_grass, after aggregating a large number of flat_fields’ feature, city_grass might be misclassified as flat_field.

(b) The effectiveness of prior knowledge embedding.

According to our definition of weights in spatial adjacency matrix, weight represents spatial correlation between neighboring node and center node.

(26) $$f_{i}^{\prime }=\overset{n}{\underset{j=0}{\sum }}a_{ij}*f_{j}$$

In Cluster-GCN, node features are aggregated in the Formula (26); $f_{i}^{\prime }$ represents updated center node I; all neighboring nodes’ feature $f_{j}$ will multiply corresponding weights $a_{ij}$ to aggregate. Node 1 (city_grass) is the center node, and the weights of all neighboring nodes are shown in Table 4. Nodes in Table 4 correspond to superpixel blocks in Figure 23.

As shown in Table 4, classes of neighbor nodes include city, road, grass, and flat_field. Features of the same class tend to be similar, so the feature aggregation of node 5 can be simplified as Formula (27).

(27) $$f_{1}^{\prime }=f_{1}+a_{12}*f_{2}+a_{13}*f_{3}+a_{14}*f_{4}+(a_{15}+a_{16}+a_{17}+a_{18}+a_{19}+a_{110})*f_{5}=f_{1}+0.5f_{2}+0.5f_{3}+0.25f_{4}+f_{5}$$

In Formula (27), $f_{1}^{\prime }$ is the feature of node 1 after aggregation, $f_{1}$ represents the original feature of node 1, $f_{2}$ represents the feature of city, $f_{3}$ represents the feature of road, $f_{4}$ represents the feature of grass, $f_{5}$ represents the feature of flat_field.

In our KGGCN model, the co-occurrence of probability between city_grass and neighboring classes can be used as prior knowledge to guide feature aggregation. Table 5 shows the comparison of weights of neighboring classes in Cluster-GCN (baseline) and KGGCN (our model).

As shown in Table 5, the weights of neighboring classes are changed after knowledge embedding. Except for city_grass itself, all neighboring classes’ weights are decreased. City decreases from 0.5 to 0.475, road from 0.5 to 0.355, grass from 0.25 to 0.07, and flat_field from 1 to 0.1. Apparently, in Cluster-GCN, during aggregation, the influence of flat_field is far more than other classes, even equal to city_grass itself, so the model will distinguish between city_grass and flat_field. However, after knowledge embedding in KGGCN, the effect of flat_field is drastically reduced, the influence of “different objects with the same spectrum” will be largely reduced, and then our model can achieve better performance.

(2) Analysis of a sample with “relation distortion”

**Figure 25:**
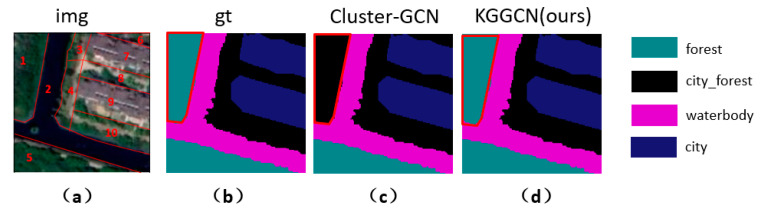
Demonstration of one sample. (**a**) is the raw remote sensing image and superpixel blocks are marked in the image; (**b**) is the ground truth of this image; (**c**) is the prediction of Cluster-GCN; (**d**) is the prediction of KGGCN.

As shown in Figure 25, node 1 is forest, classified as city_forest incorrectly in Cluster-GCN and classified as forest correctly in KGGCN. Two models both obtain correct classification for other nodes.

According to the result, we analyze the effectiveness of prior knowledge embedding.

Similar to (1) in this section, the weight distribution of all neighbor nodes is expressed in Table 6, and the comparison of weights in Cluster-GCN and KGGCN is shown in Table 7.

According to Table 6, classes of neighbor nodes include forest, city_forest, waterbody, and city.

As shown in Table 7, the weights of neighbor classes are changed after knowledge embedding. Except for forest, all neighbor classes’ weights are decreased. City forest decreases from 1.125 to 0.0495, waterbody from 0.5 to 0.1, and city from 0.25 to 0.024. As introduced before, city and city_forest are irrelevant classes to forest. In Cluster-GCN, city_forest and city belong to city scene, and they will cause the misclassification of forest. In KGGCN, the weights of city_forest and city are reduced to nearly zero.

Therefore, after knowledge embedding, the impact of irrelevant classes will be restrained, and then relevant classes will become more important. By embedding prior knowledge, “the reversal of the first law of geography” can be effectively solved.

(3) Discussion

Compared with the baseline model (Cluster-GCN), by adding the co-occurrence probability as prior knowledge into graph convolution, the KGGCN model can effectively solve the problem of “the reversal of the first law of geography”.

## 6. Supplementary Experiments of Hyperspectral Image (HSI) Classification

The main work of our KGGCN network is the semantic segmentation of high-resolution remote sensing images. To evaluate the generalization ability of the model, we have supplemented the experiment of HSI classification.

HIC is a promising but challenging task, which has been a long-researched task with wide applications such as weather forecasting, disaster prevention, and mineral exploration. It is a meaningful attempt to apply our model in HSI classification. In order to evaluate the performance of our model in HSI classification, we chose to train our model on the Indian Pines dataset.

### 6.1. Comparison of Datasets

Hyperspectral remote sensing images have a large number of bands. The spectral resolution of these images is relatively high. For example, the spectral resolution of the Indian Pines dataset is 10 nm. Because their spatial resolution is relatively low, the features of hyperspectral images are concentrated on the spectral dimension instead of spatial perspective.

The spatial resolution of high-resolution remote sensing images is generally at sub-meter level. There are several bands inside these images, of which the spectral resolution is relatively low at the same time. For example, the spectral resolution of the (Gaofen Image Dataset) GID [41] dataset is about 100 nm. That is the reason why high-resolution remote sensing images can contain abundant spatial features but relatively poor spectral features.

### 6.2. Dataset: Indian Pines Dataset

The scene is composed of 145×145 pixels and 220 spectral bands. There are 16 land-cover categories involved in this scene. Similar to methods in [42,43,44,45,46,47,48], we remove 20 water absorption channels and noise channels and keep 200 channels.

### 6.3. The Division of Training Set and Test Set

Referring to the data set division in the related methods [42,43,44,45,46,47] of hyperspectral image classification, the samples are divided into a training set and test set, as shown in Table 8. For each category, 30 labeled pixels are randomly selected for the training set. If the number of pixels in the corresponding category is less than 30, 15 pixels will be randomly selected for the training set. All the remaining pixels are used as the test set.

During training, 90% of the labeled examples are utilized to learn the network parameters, and the remaining 10% are used as validation set for hyperparameter tuning.

### 6.4. Network Training

**Figure 26:**
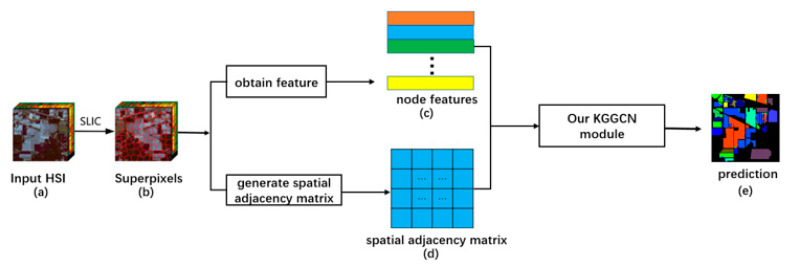
Data flow in our model. (**a**) is the original hyperspectral image. (**b**) shows the superpixels segmented by SLIC algorithm. (**c**) are node features. (**d**) is a spatial adjacency matrix. (**e**) is the prediction result of our model.

Our KGGCN model is trained on the Indian Pines dataset. Data flow in our model is shown in Figure 26.

The number of bands of the Indian Pine dataset is 220, and the spatial resolution is generally at the meter level, so the characteristic information of hyperspectral images is relatively concentrated in the spectral part. 2-D convolution in our model cannot effectively extract spatial information.

We adjusted the feature extraction method in the network structure, similar to the feature extraction method in these methods [42,43,44,45,46]. The feature of each node (i.e., superpixel) is the average spectral feature of the pixels involved in the corresponding superpixel blocks. In this case, the size of the node feature is 946×200.

Apart from obtaining node features, all other parts are the same as our methods in Section 3. Finally, the model outputs the prediction result.

### 6.5. Hyper-Parameter Settings

The KGGCN module requires two graph convolutional layers and a fully connected layer. The output dimension of the graph convolutional layer is 64, and the output dimension of the fully connected layer is 16. Batch size is set to 1, learning rate is set to 1e-30.001, and dropout is set to 0.4. The number of training rounds is 3000.

### 6.6. Experimental Results

**Figure 27:**
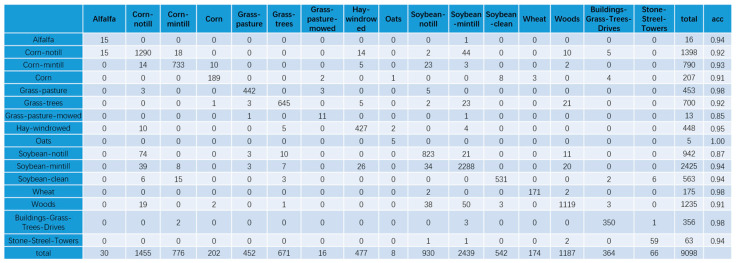
The confusion matrix of our model in test set.

The classification results of our model in the Indian Pines dataset are demonstrated in Figure 27.

Figure 27 represents the confusion matrix of our model in test set and demonstrates the accuracy of all categories. According to Figure 27, our model is less effective in classification for a few categories, such as grass-pasture-mowed and soybean–notill. For all other categories, our model achieves good performance.

**Figure 28:**
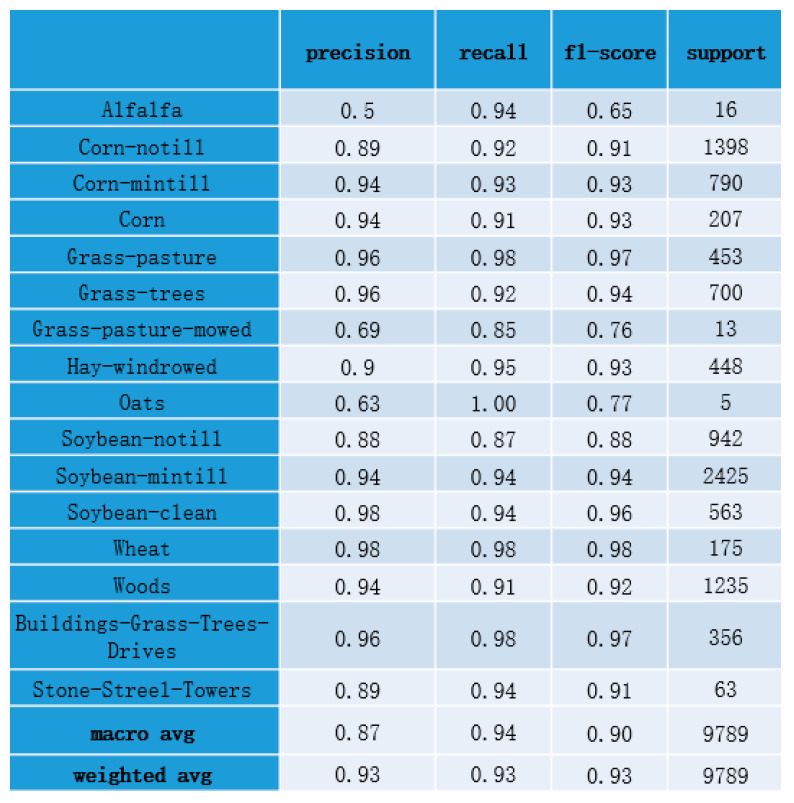
Statistical metrics of our model in test set.

Additionally, we further analyse the results in other metrics, as shown in Figure 28.

Figure 28 demonstrates statistical metrics, including precision, recall, and f1-score of each category. Macro avg in Figure 28 denotes the arithmetic average of a metric in all categories. Weighted avg in Figure 28 denotes the weighted average of a metric in all categories.

The macro avg of precision is 0.87. Precisions of alfalfa, grass-pasture-mowed, and oats are 0.5, 0.69, and 0.63, respectively, which are lower than 0.87. The reason for the poor classification effect is the small number of samples in these classes. The weighted avg of precision is 0.93. Similarly, f1-score of alfalfa, grass-pasture-mowed, and oats are 0.65, 0.76, and 0.77, respectively, which are lower than macro avg of f1-score.

All these metrics reflect the classification effect of the model for each category. As shown in Figure 28, our model is less effective in categories with small sample size, including alfalfa, grass-pasture-mowed, and oats. For other categories, our model achieves good performance in HSI classification. Then, we compared our model with other methods.

Several state-of-the-art methods are used for comparison with our model, including S^2^GCN [44], MDGCN [45], MDGCN_AGL [46], and Fast_3D_CNN [47]. The results of the Indian Pines dataset are shown in Table 9.

Table 9 demonstrates the comparison of the OA (overall accuracy), AA (average accuracy), and kappa coefficients of our model on the test set with other methods. According to the table, Fast_3D_CNN is the most efficient model in the Indian Pines dataset. The performance of our model is better than S^2^GCN and is inferior to other models.

**Figure 29:**
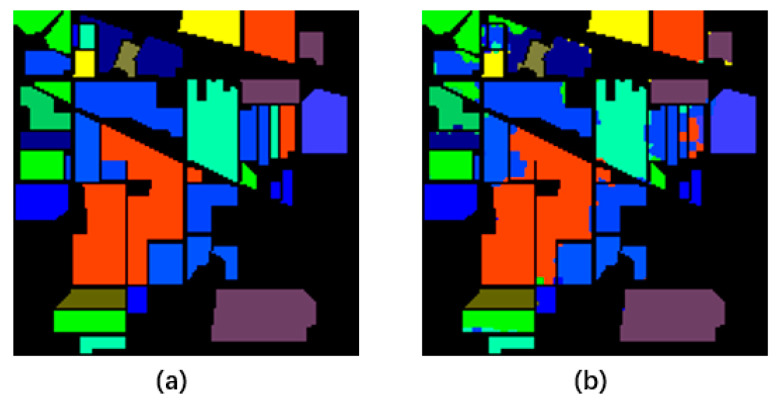
The classification result of our model. (**a**) is the ground truth; (**b**) is the classification result of our model.

Additionally, we visualized the classification result of our model, as shown in Figure 29.

In a word, our model achieves good performance in HSI classification, but at the same time, there is still room for improvement.

**Figure 30:**
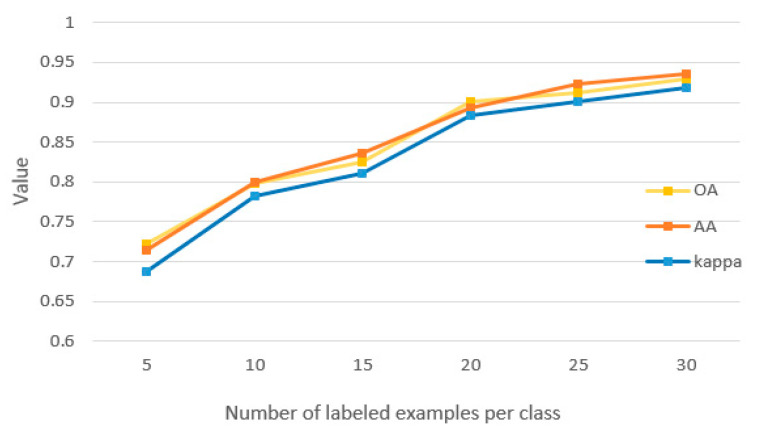
OA, AA, and kappa under different numbers of labeled examples per class.

Meanwhile, in order to check the quality of labeled training examples and their effects on final classification, we trained our model with the number of labeled samples that is set to be 5, 10, 15, 20, 25, and 30. Results are shown in Figure 30.

In Figure 30, it can be seen that the model can achieve better performance with the larger number of labeled examples.

**Figure 31:**
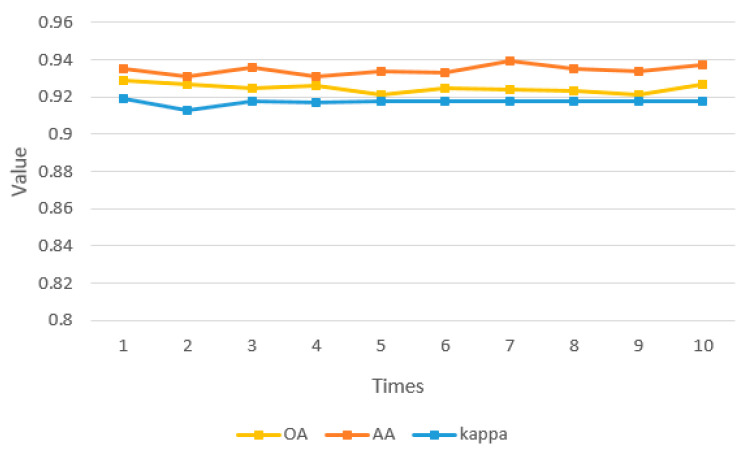
Results of 10 independent Monte Carlo trials.

To check the quantity of labeled training examples and their effects on the final result, we randomly allocated the total sample into labeled and unlabeled samples in the same proportion as the previous experiment and conducted 10 independent Monte Carlo trials, as shown in Figure 31.

In the 10 experiments, the mean values of OA, AA, and kappa are 0.925, 0.935, and 0.918, respectively, and the standard deviations are 0.0025, 0.0024, and 0.0016, respectively. These prove that the quality and quantity of labeled training examples are reasonable in our experiments, and the experimental results are stable and reliable as well.

### 6.7. Analysis of Results

We analyzed the experimental results from the following two aspects:

- **Feature extraction.** The main work of KGGCN is the semantic segmentation of high-resolution remote sensing images, where 2-D convolution is used to extract image features. However, in HSI, each pixel position contains rich spectral information, which is different from high-resolution remote sensing images. Compared with the method of feature extraction in our model, 3-D convolution has stronger capabilities for extracting the spectral information of HSI.
- **Geographic prior knowledge**. In experiments of semantic segmentation of remote sensing images, the dataset contains 1680 images, as expressed in Section 4. We can count the co-occurrence probability of each category based on all samples as geographic prior knowledge. In the HSI dataset, there is only one single image, and the co-occurrence probability of each category cannot be effectively obtained. The geographic prior knowledge is ineffective in this experiment. Thus, in HSI classification, the performance of our model is limited.

## 7. Conclusions

In this paper, to deal with the problem of insufficient application of geographic object-level semantic information (prior knowledge) and spatial correlations in semantic segmentation of remote sensing images, we propose a graph neural network model based on geo-object prior knowledge. This model uses the mechanism of prior knowledge embedding to integrate graph convolution with co-occurrence probability. Then, the node’s receptive field is extended, and the limitation of the sample context is broken through. Experimental results prove that our KGGCN model improves the pixel accuracy by almost 3.7% compared to that of Cluster-GCN, which is treated as the baseline model. The analyses of the results in Section 5 prove that the integration of prior knowledge will achieve better performance, especially in dealing with atypical samples. In addition, we evaluate our model in the HSI dataset, and the performance of our model is slightly inferior to state-of-the-art models, as shown in Section 6.

In further research, we will focus on the following aspects:

- **Scale of segmentation.** In remote sensing images, different types of geo-objects always come with different segmentation scales, so that it is important to exploit the approaches of balancing them.
- **Automatic acquisition of knowledge.** In this paper, prior knowledge is based on manual statistics and analysis, which tend to be affected by subjective factors, and they are also not efficient. To improve the method of obtaining prior knowledge, an automatic learning and adjustment method will be planned in our further research.
- **Extension of the model in HSI.** In future work, we will study the interpretation of HSI and conduct 3-D convolution to extract features. Meanwhile, we will explore the approach of integrating prior geographic knowledge with HSI.

## Figures and Tables

**Table 1:** The mIOU, Kappa, and F1-Score of the three models.

	Accuracy	mIOU	Kappa	F1-Score
U-Net	0.867	0.699	0.850	0.806
Cluster-GCN	0.871	0.769	0.872	0.855
KGGCN(ours)	**0.907**	**0.832**	**0.916**	**0.905**

**Table 2:** The pixel-based accuracies of the three models in segmentation for all classes.

	Flat_Field	Landslide	Grass	Waterbody	Village	Highway	Road	City	Terraces	Strip_Field	City_Grass	Forest	City_Forest
U-Net	0.931	0.913	0.760	0.973	0.830	0.794	0.649	0.925	0.925	0.928	0.288	0.851	0.670
Cluster-GCN	0.930	0.965	0.695	0.965	0.859	0.787	0.649	0.942	0.905	0.894	0.320	0.911	0.516
KGGCN	0.921	0.934	0.852	0.949	0.923	0.899	0.846	0.924	0.900	0.924	0.764	0.911	0.833

**Table 3:** The performance and system resource requirements of three networks.

Model	Params (M)	Mem (GB)	Flops (G)	Inf Time (FPS)
U-Net	8.64	8.85	12.60	43.01
Cluster-GCN	0.08	1.07	1.21	88.21
KGGCN (ours)	0.08	1.05	1.11	89.43

**Table 4:** Weights of all neighbor nodes.

Object	1 (City_Grass Itself)	2 (City)	3 (Road)	4 (Grass)	5 (Flat_Field)	6 (Flat_Field)	7 (Flat_Field)	8 (Flat_Field)	9 (Flat_Field)	10 (Flat_Field)
Weight	1	0.5	0. 5	0.25	0.25	0.25	0.125	0.125	0.125	0.125

**Table 5:** Comparison of weights in Cluster-GCN and KGGCN.

	City_Grass Itself	City	Road	Grass	Flat_Field
Co-occurrence probability	1	0.95	0.71	0.28	0.1
Weights in Cluster-GCN	1	0.5	0.5	0.25	1
Weights in KGGCN	1	0.475	0.355	0.07	0.1

**Table 6:** Weights of all neighbor nodes.

Object	1 (Forest Itself)	2 (Waterbody)	3 (City_Forest)	4 (City_Forest)	5 (Forest)	6 (City_Forest)	7 (City)	8 (City_Forest)	9 (City)	10 (City_Forest)
Weight	1	0.5	0.25	0.25	0.25	0.25	0.25	0.125	0.125	0.125

**Table 7:** Comparison of weights in Cluster-GCN and KGGCN.

	Forest Itself	City_Forest	Waterbody	City	Forest (Neighbor)
Co-occurrence probability	1	0.044	0.2	0.096	1
Weights in Cluster GCN	1	1.125	0.5	0.25	0.25
Weights in KGGCN	1	0.0495	0.1	0.024	0.25

**Table 8:** Indian Pines dataset samples statistics.

ID	Class	#Labeled	#Unlabeled
1	Alfalfa	30	16
2	Corn–notill	30	1398
3	Corn–mintill	30	800
4	Corn	30	207
5	Grass–pasture	30	453
6	Grass–trees	30	700
7	Grass–pasture-mowed	15	13
8	Hay–windrowed	30	448
9	Oats	15	5
10	Soybean–notill	30	942
11	Soybean–mintill	30	2425
12	Soybean–clean	30	563
13	Wheat	30	175
14	Woods	30	1235
15	Buildings–grass–trees–drives	30	356
16	Stone–stell–towers	3	63

**Table 9:** The comparison of models in Indian Pines dataset.

	OA	AA	Kappa
**S^2^GCN**	0.8849	0.9299	0.8800
**MDGCN**	0.9347	0.9624	0.9255
**MDGCN-AGL**	0.9466	0.9537	0.9392
**Fast_3D_CNN**	0.9775	0.9454	0.9744
**KGGCN (ours)**	0.9294	0.9350	0.9190

## Data Availability

The data presented in this study are available on request from the corresponding author. The data are not publicly available due to privacy reasons.
